# Clinical implications of CD4^+^ T cell subsets in adult atopic asthma patients

**DOI:** 10.1186/s13223-018-0231-3

**Published:** 2018-03-02

**Authors:** Matthew Wiest, Katherine Upchurch, Wenjie Yin, Jerome Ellis, Yaming Xue, Bobby Lanier, Mark Millard, HyeMee Joo, SangKon Oh

**Affiliations:** 1 Baylor Institute for Immunology Research, 3434 Live Oak St., Dallas, TX 75204 USA; 2 Institute for Biomedical Studies, Baylor University, Waco, TX USA; 3 Texas Allergy Experts, Fort Worth, TX USA; 4 Martha Foster Lung Care Center, Baylor University Medical Center, Dallas, TX USA

**Keywords:** Asthma, Atopic, CD4^+^ T cell, CCR7, CCR4, Integrin, Alpha 4, CTLA-4, β-Agonist, Corticosteroid, Therapy

## Abstract

**Background:**

T cells play a central role in chronic inflammation in asthma. However, the roles of individual subsets of T cells in the pathology of asthma in patients remain to be better understood.

**Methods:**

We investigated the potential signatures of T cell subset phenotypes in asthma using fresh whole blood from adult atopic asthma patients (n = 43) and non-asthmatic control subjects (n = 22). We further assessed their potential clinical implications by correlating asthma severity.

**Results:**

We report four major features of CD4^+^ T cells in the blood of atopic asthma patients. First, patients had a profound increase of CCR7^+^ memory CD4^+^ T cells, but not CCR7^−^ memory CD4^+^ T cells. Second, an increase in CCR4^+^ CD4^+^ T cells in patients was mainly attributed to the increase of CCR7^+^ memory CD4^+^ T cells. Accordingly, the frequency of CCR4^+^CCR7^+^ memory CD4^+^ T cells correlated with asthma severity. Current common asthma therapeutics (including corticosteroids) were not able to affect the frequency of CCR4^+^CCR7^+^ memory CD4^+^ T cell subsets. Third, patients had an increase of Tregs, as assessed by measuring CD25, Foxp3, IL-10 and CTLA-4 expression. However, asthma severity was inversely correlated only with the frequency of CTLA-4^+^ CD4^+^ T cells. Lastly, patients and control subjects have similar frequencies of CD4^+^ T cells that express CCR5, CCR6, CXCR3, CXCR5, CD11a, or α4 integrin. However, the frequency of α4^+^ CD4^+^ T cells in patients correlated with asthma severity.

**Conclusions:**

CCR4^+^CCR7^+^ memory, but not CCR4^+^CCR7^−^ memory, α4^+^, and CTLA4^+^ CD4^+^ T cells in patients show significant clinical implications in atopic asthma. Current common therapeutics cannot alter the frequency of such CD4^+^ T cell subsets in adult atopic asthma patients.

**Electronic supplementary material:**

The online version of this article (10.1186/s13223-018-0231-3) contains supplementary material, which is available to authorized users.

## Background

Chronic inflammation in the lung with airway hyper-responsiveness is one of the major characteristics of asthma [1]. Asthma is a highly heterogeneous disease comprised of distinct clinical, immunological, and genetic phenotypes [2–4]; however, the pathogenesis of asthma has been classically characterized as elevated Th2-type inflammatory responses to antigen. These elevated Th2-type cells have also been found in the blood of asthma patients, indicating that immune cells responsible for chronic inflammation in the lung circulate in the blood [5–8].

The normal response to a harmless allergen is tolerance, but asthmatic patients can respond with elevated Th2-type immune responses. Th2-type CD4^+^ T cells secrete IL-4, IL-5, and IL-13, which play important downstream roles in asthma pathogenesis [9]. IL-4 induces IgE class-switching and expression of vascular cell adhesion molecule-1 on endothelial cells [10, 11]. IL-5 is crucial for the activation of eosinophils and their migration into the lung [12]. IL-13 is associated with various important events during the effector phase of asthma including airway hyper-responsiveness, mucus hyper-production, and airway remodeling [13, 14]. However, the high level of clinical heterogeneity of asthma suggests that the pathogenesis of asthma must not be solely driven by Th2-type immune responses [15]. In almost all patients with asthma, one can find a counter-regulatory population, regulatory T cells (Tregs), that are capable of suppressing inflammatory responses [16–18]. In addition, CD8^+^ T cells can also contribute to the etiopathology of asthmatic inflammation [19, 20]. Overall, T cells can play a central role in the initiation, progression, and exacerbation of asthma. However, the underlying mechanisms of the chronic inflammation in the lung and the levels of contribution by different T cell subsets remain to be fully elucidated.

Antigen-experienced T cells are phenotypically classified into effector and memory T cell populations, the latter being subdivided into CCR7^−^ effector memory T cells (Tem) and CCR7^+^ central memory T cells (Tcm) [21]. It has been previously reported that memory T cells are associated with chronic inflammatory diseases [22, 23]. However, the specific subpopulations of human memory T cells that are responsible for chronic allergic disorders, including asthma, have not been well characterized. This is partly due to variations in the phenotypes of pathogenic T cells in asthma patients. It is further exacerbated by patient-intrinsic factors, such as differences in offending allergens, as well as environmental changes, which can affect timing of allergen exposure (e.g., perennial vs. seasonal allergy). Furthermore, the number of memory T cells recoverable from lungs of asthma patients is extremely limited. Despite these complicating factors, it is imperative to find which T cell subsets, especially which subset of memory T cells, are associated with chronic inflammation in the lungs of asthma patients.

To this end, we hypothesized that T cells in atopic asthma patients display unique phenotypes and functions that can support chronic inflammation in the lung. We utilized fresh whole blood from atopic asthma patients and non-asthmatic control subjects as a source of T cells for investigation. Although T cells in the peripheral blood may not be the same as those in the lungs of asthma patients, their altered phenotypes and functions could also be associated with the pathogenesis of asthma [23–27]. We found that T cells in the blood of adult atopic asthma patients display several unique phenotypic and functional features. More importantly, some of the new features found in this study correlate with asthma severity, supporting the clinical relevance of these altered phenotypes and functions in atopic asthma patients. Further clinical data analysis concluded that corticosteroids do not affect these altered phenotypes or functions of T cells in atopic asthma. Data from this study could thus help us extend our knowledge of the pathophysiology of human asthma and potentially contribute to the rational design of new therapeutic approaches for asthma in the future.

## Methods

### Patients and control subjects

Adult asthma patients (n = 43) were recruited in this study (Table 1). Clinical variables, including asthma control test (ACT) score, lung function, as defined by the forced expiratory volume in 1 s (% predicted FEV1), and frequency of symptoms (e.g., total number of symptoms per week and nighttime sleep disruptions) as defined by the expert panel report from the National Asthma Education and Prevention Program [28], were acquired. All patients showed positive responses to at least one allergen by a skin prick test, as measured by the assessment of hypersensitivity (wheal—a raised white bump surrounded by a small circle of itchy red skin) to allergens. Except for four patients, all patients were being treated with either short- or long-acting β-agonists at the time of blood draw. Non-asthmatic control subjects (n = 22) were also recruited. All subjects were enrolled under protocols approved by the Institutional Review Board of Baylor Scott & White Research Institute. Donors were excluded if they were pregnant, under the age of 18, or if they had any other chronic diseases.

**Table 1:** Information of atopic asthma patients and non-asthmatic control subjects recruited in this study

Characteristics	Asthma patients	Non-asthmatic controls
Total population, n	43	22
Inhaled corticosteroid (%)	25 (58)	
Oral corticosteroid (%)	7 (14)	
Leukotriene inhibitor (%)	22 (51)	
Untreated (%)	9 (21)	
Age (years)	51.9 (± 11.31)	47.59 (± 12.60)
Sex (M/F) (%M)	15/28 (35)	8/14 (36)
Height (in)	66.54 (± 3.31)	67.55 (± 3.41)
Weight (lbs)	188.8 (± 39.30)	162.0 (± 28.99)
Caucasian, n (%)	38 (88)	17 (77)
African American, n (%)	3 (7)	4 (18)
Asian, n (%)	1 (2)	1 (5)

All data are expressed as mean with SD (if applicable)

### Whole blood T cell analysis

Blood was drawn twice over a 1-week interval and average values from two separate experiments were used. Complete blood count (CBC) was performed with Coulter Ac·T™ 5diff (Beckman Coulter). Whole blood (200 μL) was stained with the indicated antibodies and 50 μL of brilliant stain buffer (Becton–Dickinson: BD) to enhance brilliant violet fluorochrome stability. Red blood cells were lysed, and cells were fixed with lysing solution (BD). Stained cells were analyzed on an LSR Fortessa flow cytometer (BD), and the results were analyzed with Flow Jo (TreeStar). Detailed information for antibodies used in this study is summarized in Additional file 1: Table S1. To count cell numbers, 20 μL of CountBright absolute counting beads (Life Technologies) were added to each sample. Cell counts were calculated using the number of cell events (A) divided by the number of bead events (B) multiplied by the assigned number of counting beads added based on lot (C) divided by the volume of the sample (D):

$$\frac{\text{A}}{\text{B}}\; \times \;\frac{\text{C}}{\text{D}}\; = \;{\text{concentration }}\;{\text{of}}\;{\text{sample}} .$$

### PBMC isolation and measurement of T cell cytokines

Peripheral blood mononuclear cells (PBMCs) were isolated by density gradient centrifugation using Ficoll-Plaque PLUS (GE Healthcare). PBMCs were plated at 5 × 10^5^ cells/100 μL in 96-well U-bottom plates in RPMI 1640 (Invitrogen) supplemented with HEPES (Invitrogen), 1% non-essential amino acids, 2 mM l-glutamate (Sigma-Aldrich), 50 μg/mL penicillin, and 50 μg/mL streptomycin (Life Technologies). T cells were stimulated with αCD3/αCD28 human dynabeads (Life Technologies) at a 1:1, bead:cell ratio. The amounts of cytokines in diluted supernatants were measured by multiplex bead-based assay (Bio-Rad) after 36-h stimulation when the amount of cytokines reaches maximum levels. A 5-parameter curve fitting algorithm was applied for standard curve generation. Detection limits of the standard curve were 0.2 ng/mL < IL-10 ≤ 3.5 ng/mL, 0.2 ng/mL ≤ IFN-γ ≤ 15 ng/mL, 0.05 ng/mL ≤ IL-4 ≤ 5 ng/mL, 0.2 ng/mL ≤ IL-5 ≤ 1 ng/mL, and 0.2 ng/mL ≤ IL-13 ≤ 3 ng/mL. For intracellular staining, cells were stimulated with αCD3/αCD28 dynabeads for 5–6 h, with Golgiplug (BD) added 1–2 h after stimulation.

### Statistical analysis

Statistical significance was determined using a non-parametric Mann–Whitney test. One-way analysis of variance (ANOVA) with the Tukey test was utilized for statistical significance where specified. Correlation analysis was performed with non-parametric Spearman correlation. Statistical significance analysis was performed with Prism 5 (GraphPad Software). Significance was set at P < 0.05.

## Results

### Atopic asthma patients have an increase of CCR7^+^ memory CD4^+^ T cells

**Fig. 1:**
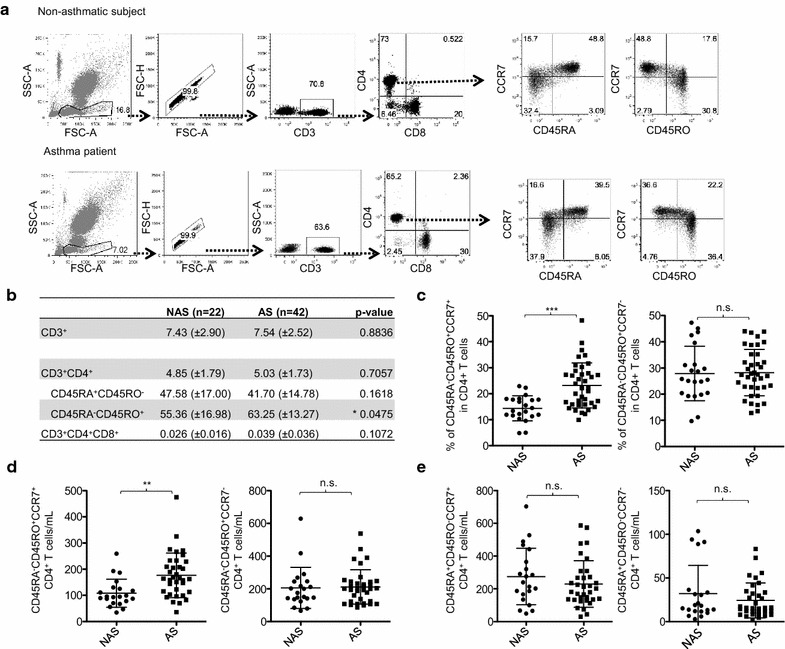
Altered distribution of memory CD4^+^ T cells in patients with atopic asthma. **a** T cell gating strategy. Cells were gated based on isotype control antibody staining. **b** Average percentages with standard deviation of T cells (CD3^+^), CD4^+^ T cells, and CD4^+^CD8^+^ T cells. CD45RA^+^ and CD45RO^+^ are quantified as percent of CD3^+^CD4^+^ T lymphocytes. **c** Percentage of CD45RA^−^CD45RO^+^CCR7^+^ (left) and CD45RA^−^CD45RO^+^CCR7^−^ (right) T cells in CD4^+^ T cells in non-asthmatic subjects (NAS) and asthma patients (AS). **d** Number of CD45RA^−^CD45RO^+^CCR7^+^CD4^+^ (left) and CD45RA^−^CD45RO^+^CCR7^−^CD4^+^ (right) T cells/µL of blood in NAS and AS. **e** Percentage of CD45RA^+^CD45RO^−^CCR7^−^ (left) and CD45RA^+^CD45RO^−^CCR7^+^ (right) in CD4^+^ T cells in NAS and AS. Statistical tests were performed with non-parametric Mann–Whitney test. *P < 0.05, **P < 0.01, ***P < 0.001, n.s.: not significant. Error bars indicate SD

We first investigated the frequency of naïve and memory T cells in the peripheral blood of adult atopic asthma patients (n = 43) and non-asthmatic control subjects (n = 22) by staining whole blood with antibodies specific for surface molecules (Fig. 1a). As summarized in Fig. 1b, atopic asthma patients and control subjects had similar percentages of CD3^+^, CD3^+^CD4^+^, CD3^+^CD4^+^CD45RA^+^CD45RO^−^ and CD3^+^CD4^+^CD8^+^ T cells in their blood. However, atopic asthma patients had a greater percentage of CD45RA^−^CD45RO^+^ memory CD4^+^ T cells than control subjects, as previously described [29]. CBC data show that total numbers of lymphocytes were not significantly different in the two groups (Table 2). However, atopic asthma patients had significantly more circulating eosinophils and neutrophils. Basophil numbers were also increased in patients, but the difference was not statistically significant. No significant difference was observed for red blood cell or platelet counts in the two groups.

**Table 2:** Complete blood counts, per microliter of blood

Cell types	Atopic asthma patients	Non-asthmatic controls	*p*-value
Lymphocytes	1473 (± 389)	1353 (± 332)	0.2347
Eosinophils	321 (± 258)	105 (± 38)	0.0005
Neutrophils	3953 (± 1417)	2310 (± 551)	0.0001
Basophils	33 (± 17)	25 (± 13)	0.0529
Monocytes	577 (± 207)	410 (± 233)	0.0057
RBC	4.03 × 10^6^ (± 0.442 × 10^6^)	3.93 × 10^6^ (± 0.343 × 10^6^)	0.3938
Platelets	215 × 10^3^ (± 69.1 × 10^3^)	215 × 10^3^ (± 56.5 × 10^3^)	0.9863

Data are expressed as mean cell number per microliter with SD. A Bonferroni correction was used for the multiple comparisons

Memory (CD45RA^−^CD45RO^+^) CD4^+^ T cells were further analyzed based on their CCR7 expression (Fig. 1a). Compiled data from atopic asthma patients and control subjects indicated that atopic asthma patients have more CD45RA^−^CD45RO^+^CCR7^+^ CD4^+^ T cells than control subjects (Fig. 1c, left). The percentage of CD45RA^−^CD45RO^+^CCR7^−^ CD4^+^ T cells was similar in the two groups (Fig. 1c, right). The numbers of CD45RA^−^CD45RO^+^CCR7^+^ (Fig. 1d, left) and CD45RA^−^CD45RO^+^CCR7^−^ CD4^+^ T cells per microliter of blood (Fig. 1d, right) also showed similar trends. There was no significant correlation between ages of patients recruited in this study and the frequency of CD45RA^−^CD45RO^+^, CD45RA^−^CD45RO^+^CCR7^+^, or CD45RA^−^CD45RO^+^CCR7^−^ CD4^+^ and CD8^+^ T cells (Additional file 2: Figure S1), confirming previously reported observations [30, 31]. Both atopic asthma patients and control subjects had similar frequencies of naïve (CD45RA^+^CD45RO^−^CCR7^+^) and CD45RA^+^CD45RO^−^CCR7^−^ CD4^+^ T cells (Fig. 1e).

Therefore, we concluded that adult atopic asthma patients have an increase of circulating CD45RA^−^CD45RO^+^CCR7^+^ T cells, but not CD45RA^−^CD45RO^+^CCR7^−^ or CD45RA^+^CD45RO^−^ CD4^+^ T cells.

### The increase of CCR7^+^ memory CD4^+^ T cells is observed in atopic asthma subgroups and is resistant to common therapeutics

**Fig. 2:**
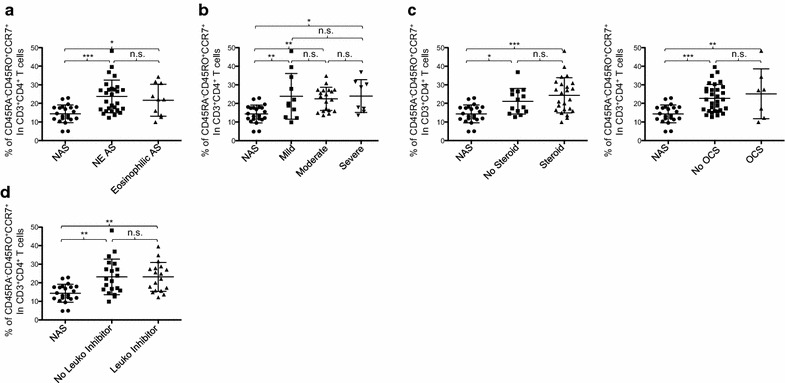
The increase of CCR7^+^ memory CD4^+^ T cells is unaffected by common therapeutics. **a** Percentage of CD45RA^−^CD45RO^+^CCR7^+^ cells in CD3^+^CD4^+^ T cells across non-asthmatic subjects (NAS) and asthma patients (AS) with (Eosinophilic AS) or without (NE AS) blood eosinophilia. **b** Percentage of CD45RA^−^CD45RO^+^CCR7^+^ cells in CD4^+^ T cells in atopic asthma of different severities based on % predicted FEV1 scores (mild: > 80%, moderate: 60–79%, severe: < 60%). Percentage of CD45RA^−^CD45RO^+^CCR7^+^ cells in CD3^+^CD4^+^ T cells in NAS and AS treated with/without corticosteroids (**c**, left) oral corticosteroid (OCS) (**c**, right) and leukotriene inhibitor (**d**). Analysis based on one-way ANOVA with Tukey test. *P < 0.05, **P < 0.01, ***P < 0.001, n.s.: not significant. Error bars indicate SD

Due to the heterogeneity of asthma phenotypes and clinical variation, we next investigated whether the increase of CCR7^+^ memory CD4^+^ T cells is a common feature of different asthma subtypes. Atopic asthma patients were divided into two subgroups, based on their blood eosinophil counts. We found that patients with blood eosinophilia (eosinophil count > 450/µL blood) and non-eosinophilia (eosinophil count < 450/µL blood) had similar percentages of circulating CCR7^+^ memory CD4^+^ T cells, but both subgroups of patients had a higher percentage of CCR7^+^ memory CD4^+^ T cells than non-asthmatic control subjects (Fig. 2a). We next divided patients into three subgroups based on their % predicted FEV1 (mild: FEV1 > 80; moderate: FEV1 = 60–80; and severe: FEV1 < 60). As shown in Fig. 2b, all three subgroups of patients had higher percentages of CCR7^+^ memory T cells than control subjects. There was no significant difference between the three subgroups of patients.

We further investigated whether prescribed therapeutics could impact the frequency of the CCR7^+^ memory CD4^+^ T cells. Patients treated with corticosteroids [either inhaled (N = 26) or oral corticosteroid (N = 7)] and patients who did not receive corticosteroid therapy (N = 14) within 2 weeks before blood draw had more CCR7^+^ memory CD4^+^ T cells than non-asthmatic control subjects (Fig. 2c, left). However, the two groups of patients (corticosteroid versus no corticosteroid) had similar percentages of CCR7^+^ memory CD4^+^ T cells. We further found that oral corticosteroid usage did not significantly alter the percentage of such CD4^+^ T cell subset (Fig. 2c, right). In addition, inhaled corticosteroid did not significantly alter the percentage of CCR7^+^ memory CD4^+^ T cells in patients (data not shown). Furthermore, leukotriene inhibitors did not alter the percentage of CCR7^+^ memory CD4^+^ T cells in the blood of adult atopic asthma patients (Fig. 2d).

We thus concluded that the increase of circulating CCR7^+^ memory CD4^+^ T cells is a common feature of adult atopic asthma patients. This feature was also maintained throughout different atopic asthma subtypes examined in this study. In addition, corticosteroids or leukotriene inhibitors were not able to change the frequency of CCR7^+^ memory CD4^+^ T cells in patients.

### Atopic asthma patients have an increase of CCR4^+^ CD4^+^ T cells, but this is mainly due to the increase of CCR7^+^ memory CD4^+^ T cells

Chemokine receptors expressed on T cells can contribute to T cell migration into local tissues as well as into lymph nodes. They are also indicative markers of T cell subsets—CXCR3 for Th1, CCR4 for Th2, CCR6 for Th17, and CXCR5 for follicular helper T cells (Tfh) [32]. CD4^+^ T cells expressing CCR4 have thus been of particular interest in the pathogenesis of allergic asthma. As such, we analyzed the frequency of CD4^+^ T cell subsets expressing CCR4 in atopic asthma patients and non-asthmatic control subjects.

**Fig. 3:**
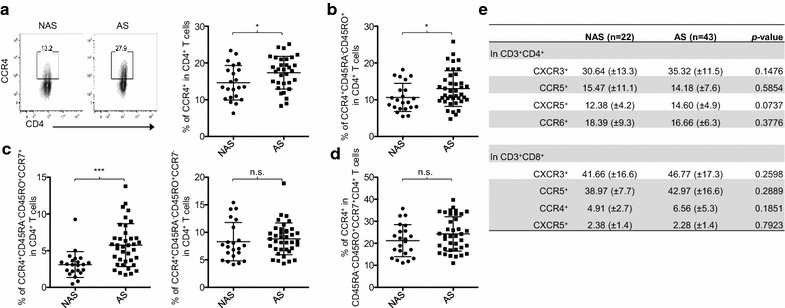
Atopic asthma patients have an increase of CCR4^+^CCR7^+^ memory CD4^+^ T cells. **a** Representative FACS staining of CCR4 in CD3^+^CD4^+^ T cells and compiled percentages in non-asthmatic subjects (NAS) and asthma patients (AS). **b** Percentage of CD45RA^−^CD45RO^+^CCR4^+^ cells in CD3^+^CD4^+^ T cells in NAS and AS. **c** Percentage of CCR4^+^CD45RA^−^CD45RO^+^CCR7^+^ and CCR4^+^CD45RA^−^CD45RO^+^CCR7^−^ cells in CD3^+^CD4^+^ T cells in NAS and AS. **d** Percentage of CCR4^+^ cells in CCR7^+^ memory CD4^+^ T cells. Data represent average percentages with SD. Cells were gated based on isotype antibody staining. **e** Average percentages with standard deviation of chemokine receptors on CD4^+^ T cells and CD8^+^ T cells. Significance was determined with a non-parametric Mann–Whitney test. *P < 0.05, **P < 0.01, ***P < 0.001, n.s.: not significant. Error bars indicate SD

Atopic asthma patients have a higher percentage of CCR4^+^CD4^+^ T cells (Fig. 3a), as has been previously described [33]. Such increases can also be seen in the percentage of CCR4^+^CD45RA^−^CD45RO^+^ CD4^+^ T cells (Fig. 3b). However, the difference between patients and control subjects was more significant when analyzing the percentage of CCR4^+^CD45RA^−^CD45RO^+^CCR7^+^ cells in CD4^+^ T cells (Fig. 3c, left). There was no significant difference in the percentage of CCR4^+^CD45RA^−^CD45RO^+^CCR7^−^ cells in CD4^+^ T cells between the two groups (Fig. 3c, right), which was consistent with the data in Fig. 1c. Interestingly, there was no significant difference in the percentage of CCR4^+^ cells in CCR7^+^ memory CD4^+^ T cells between the two groups (Fig. 3d). This indicates that the increase of CCR4^+^CD45RA^−^CD45RO^+^CCR7^+^ cells in CD4^+^ T cells (Fig. 3c) was mainly due to the increase of CCR7^+^ memory cell in CD4^+^ T cells in patients.

No significant difference was seen in CXCR5^+^, CXCR3^+^, CCR5^+^, or CCR6^+^ CD4^+^ T cells in the two groups (Fig. 3e). There was no significant difference of CXCR3^+^, CXCR5^+^, CCR4^+^, or CCR5^+^ CD8^+^ T cells in the two groups.

We thus concluded that adult atopic asthma patients have increased CCR4^+^ CD4^+^ T cells in their blood and this increase is mainly due to the increase of CCR4^+^CD45RA^−^CD45RO^+^CCR7^+^ CD4^+^ T cells.

### The frequency of CCR4^+^ CD4^+^ T cells varies among atopic asthma subtypes, but it is not influenced by corticosteroid or leukotriene treatment

**Fig. 4:**
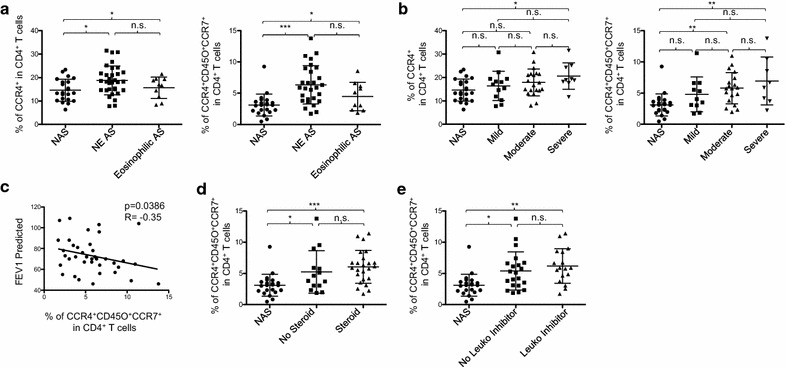
Frequency of CCR4^+^CCR7^+^ memory CD4^+^ T cell is unperturbed by corticosteroid therapy. **a** Percentage of CCR4^+^ cells (left) and CCR4^+^CD45RA^−^CD45RO^+^CCR7^+^ cells in CD4^+^ T cells (right) in non-asthmatic subjects (NAS) and asthma patients (AS) (Eosinophilic AS and NE AS). **b** Percentage of CCR4^+^ (left) and CCR4^+^CD45RA^−^CD45RO^+^CCR7^+^ cells (right) in CD4^+^ T cells in atopic asthma of different severities based on % predicted FEV1 scores (mild: > 80%, moderate: 60–79%, severe: < 60%). **c** Non-parametric Spearman correlation analysis of percentage of CCR4^+^CD45RA^−^CD45RO^+^CCR7^+^ cells in CD4^+^ T cells with % predicted FEV1 scores of AS. **d**, **e** Percentage CCR4^+^CD45RA^−^CD45RO^+^CCR7^+^ cells in CD4^+^ T cells in NAS and AS treated with/without corticosteroids (**d**) and leukotriene inhibitor (**e**). Analysis based on one-way ANOVA with Tukey test. *P < 0.05, **P < 0.01, ***P < 0.001, n.s.: not significant. Error bars indicate SD

We next investigated whether the increase of circulating CCR4^+^ CD4^+^ T cells in patients could be a feature of asthma subtypes. Patients without blood eosinophilia showed a significantly higher percentage of CCR4^+^ CD4^+^ T cells (Fig. 4a, left). This difference was even more significant when the percentage of CCR4^+^CD45RA^−^CD45RO^+^CCR7^+^ CD4^+^ T cells was compared (Fig. 4a, right). Patients with blood eosinophilia did not show a significant increase of either CCR4^+^ CD4^+^ or CCR4^+^CD45RA^−^CD45RO^+^CCR7^+^ CD4^+^ T cells compared to control subjects.

The percentage of CCR4^+^ in CD4^+^ T cells was significantly different between control subjects and patients with severe asthma based on the % predicted FEV1 (Fig. 4b, left). Patients with moderate to mild asthma did not show such difference. However, both moderate and severe patient subgroups had significantly greater percentages of CCR4^+^CD45RA^−^CD45RO^+^CCR7^+^ CD4^+^ T cells than control subjects (Fig. 4b, right). Furthermore, the percentages of CCR4^+^ CD45RA^−^CD45RO^+^CCR7^+^ CD4^+^ T cells (Fig. 4c) inversely correlated with % predicted FEV1 scores of all patients. We also found that the increase of CCR4^+^CD45RA^−^CD45RO^+^CCR7^+^ in CD4^+^ T cells was not significantly influenced by either corticosteroid (Fig. 4d) or leukotriene inhibitor treatment (Fig. 4e).

Taken together, we concluded that the frequency of CCR4^+^ CD4^+^ T cells, particularly CCR4^+^CD45RA^−^CD45RO^+^CCR7^+^ CD4^+^ T cells, varies among atopic asthma subtypes, but are not significantly influenced by corticosteroid or leukotriene inhibitor treatments. In addition, the percentage of circulating CCR4^+^CD45RA^−^CD45RO^+^CCR7^+^ CD4^+^ T cells in patients correlated with asthma severity, as measured with % predicted FEV1 scores. Either corticosteroid or leukotriene inhibitor treatment did not alter the percentages of CD4^+^ T cells expressing the chemokine receptors tested in Fig. 3f (data not shown).

### Increase of CRTH2^+^ CD4^+^ T cells in patients is also due to the increase of CCR7^+^ memory CD4^+^ T cells

CRTH2 is expressed on eosinophils, basophils and mast cells as well as Th2-type memory CD4^+^ T cells [34] and is also known to be associated with the pathogenesis of asthma [35]. Palikhe et al. have also reported that the increase of circulating CRTH2^+^ CD4^+^ T cells is a feature of severe asthma [5]. However, another study reported that CRTH2^+^ CD4^+^ T cells are unlikely to have a significant involvement in the pathogenesis of asthma in patients, although asthma patients have increased CRTH2^+^ CD4^+^ T cells in both blood and BAL fluid [36]. We thus investigated CRTH2 expression on the subsets of memory CD4^+^ T cells and further tested whether the frequency of the subsets of CRTH2^+^ memory CD4^+^ T cells is associated with any clinical readouts for assessing disease severity, including % predicted FEV1 scores.

**Fig. 5:**
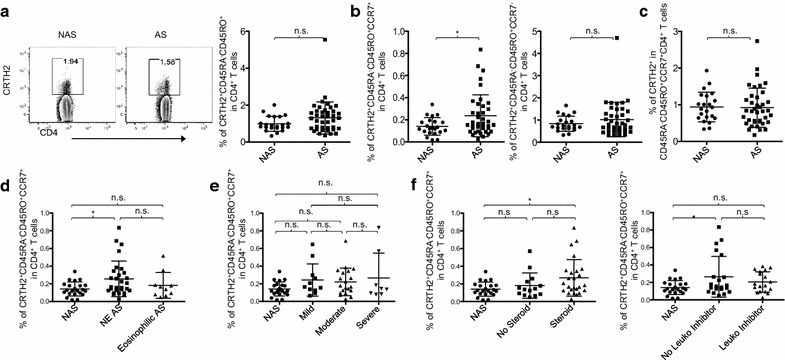
CRTH2-expressing CCR7^+^ memory CD4^+^ T cells are increased in atopic asthma patients. **a** Representative FACS staining of CRTH2 in CD3^+^CD4^+^ (left) and percentage of CRTH2^+^CD45RA^−^CD45RO^+^ cells in CD4^+^ T cells in non-asthmatic subjects (NAS) and asthma patients (AS). Gating strategy based on isotype antibody staining. **b** Percentage of CRTH2^+^CD45RA^−^CD45RO^+^CCR7^+^ cells (left) and CRTH2^+^CD45RA^−^CD45RO^+^CCR7^−^ cells (right) in CD4^+^ T cells of NAS and AS patients. **c** Percentage of CRTH2^+^ cells in CCR7^+^ memory CD4^+^ T cells of NAS and AS. **d** Percentage of CRTH2-expressing CCR7^+^ memory CD4^+^ T cells from NAS and AS (Eosinophilic AS and NE AS). **e** Percentage of CRTH2-expressing CCR7^+^ memory CD4^+^ T cells in NAS and across asthma severities defined by % predicted FEV1 scores (mild: > 80%, moderate: 60–79%, severe: < 60%). **f** Percentage of CRTH2-expressing CCR7^+^ memory CD4^+^ T cells in NAS and AS treated with/without corticosteroids (left) and leukotriene inhibitor (right) *P < 0.05, n.s.: not significant. Errors bars indicate SD

We found that atopic asthma patients and control subjects had similar percentages of CRTH2^+^ cells in CD4^+^ T cells (Fig. 5a, left) and CRTH2^+^CD45RO^+^ memory cells in CD4^+^ T cells (Fig. 5a, right). However, patients had an increase of CRTH2^+^CD45RA^−^CD45RO^+^CCR7^+^ CD4^+^ T cells (Fig. 5b, left) without an increase of CRTH2^+^CD45RA^−^CD45RO^+^CCR7^−^ CD4^+^ T cells (Fig. 5b, right). There was no significant difference in the percentage of CRTH2^+^ cells in CD45RA^−^CD45RO^+^CCR7^+^ CD4^+^ T cells (Fig. 5c). Therefore, the increase of CRTH2^+^CD45RA^−^CD45RO^+^CCR7^+^ CD4^+^ T cells in patients was mainly due to the increase of CCR7^+^ memory CD4^+^ T cells, which is in line with the data in Figs. 1, 2, 3, and 4.

Consistent with the higher percentage of CCR4^+^CD45RA^−^CD45RO^+^CCR7^+^ CD4^+^ T cells in patients without blood eosinophilia (Fig. 4a), atopic asthma patients without blood eosinophilia showed an increase of CRTH2^+^CD45RA^−^CD45RO^+^CCR7^+^ CD4^+^ T cells, compared to control subjects (Fig. 5d). In addition, the two groups of patients had similar percentages of circulating CRTH2^+^CD45RA^−^CD45RO^+^CCR7^+^ CD4^+^ T cells. Although patients had a higher percentage of CRTH2^+^CD45RA^−^CD45RO^+^CCR7^+^ cells in CD4^+^ T cells (Fig. 5b), such an increase was not seen when patients were divided into three subgroups based on the % predicted FEV1 scores (Fig. 5e). Furthermore, percentages of CRTH2^+^CD45RA^−^CD45RO^+^CCR7^+^ cells in CD4^+^ T cells or numbers of CRTH2^+^CD45RA^−^CD45RO^+^CCR7^+^ CD4^+^ T cells did not correlate with % predicted FEV1 scores or any other clinical variables, including ACT scores, FVC1%, FEV1/FVC1, frequency of β-agonist usages and night-time awakenings (data not shown). Data in Fig. 5f show that patients received corticosteroid therapy (left) and patients who were not treated with leukotriene inhibitor (right) have increased proportions of CRTH2^+^CD45RA^−^CD45RO^+^CCR7^+^ cells when compared to control subjects. Corticosteroid (Fig. 5f, left) treatments did not alter the proportion of CRTH2^+^CD45RA^−^CD45RO^+^CCR7^+^ cells in patients.

We thus concluded that adult atopic asthma patients had a minor increase of circulating CRTH2^+^ CD4^+^ T cells and this increase was also mainly due to the increase of central memory CD4^+^ T cells. However, such an increase in CRTH2^+^CD45RA^−^CD45RO^+^CCR7^+^ CD4^+^ T cells was not significantly associated with asthma severity and was not affected by corticosteroid treatment.

### Patients have an increase of CD4^+^ Tregs, but only CTLA-4^+^ CD4^+^ T cells show clinical relevance

Tregs exist in both healthy people and asthma patients [16, 37, 38]. The balance between Tregs and inflammatory Th2-type T cells could be a critical factor that determine immune tolerance or inflammation in the lung. Previous studies reported that Tregs in asthma patients might not be fully functional [37–39]. However, another study showed that such Tregs can suppress allergic inflammatory responses [16]. To gain better insight into Tregs in adult atopic asthma patients we assessed the frequency of Tregs along with those expressing IL-10. We further tested whether the frequency of Tregs in patients was clinically relevant.

**Fig. 6:**
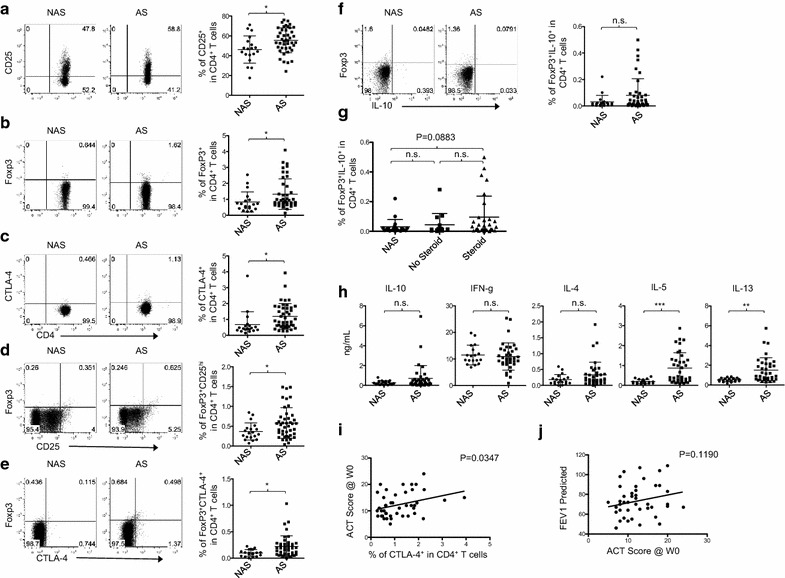
Frequency of CTLA4^+^ T cells in atopic asthma patients inversely correlates with clinical severity. Representative FACS staining and summarized data for the frequency of **a** CD25^+^, **b** Foxp3^+^, **c** CTLA-4^+^, **d** Foxp3^+^CD25^hi^ and **e** Foxp3^+^CTLA-4^+^ in live CD4^+^ T cells. **f** PBMCs were stimulated with αCD3/αCD28 beads at a 1:1 bead:cell ratio for 5–6 h in the presence of GolgiPlug. Representative FACS staining (left) and summarized frequency of Foxp3^+^IL-10^+^ in CD4^+^ T cells after stimulation (right). Gating strategy based on isotype antibody staining profile. **g** Analysis of frequency of Foxp3^+^IL-10^+^ in CD4^+^ T cells from non-asthmatic subjects (NAS) and asthma patients (AS) treated with/without corticosteroid. **h** Cytokines in the supernatants of NAS and AS T cells cultured for 36 h in the presence of αCD3/αCD28-coated beads. **i** Correlation analysis of frequency of CTLA-4-expressing CD4^+^ T cells in asthma patients with ACT scores. **j** Correlation analysis of % predicted FEV1 and ACT scores. *P < 0.05. Errors bars indicate SD

As shown in Fig. 6a, atopic asthma patients have significantly more CD25^+^ CD4^+^ T cells than non-asthmatic control subjects. In addition, significant increases of Foxp3^+^ (Fig. 6b) and CTLA-4^+^ CD4^+^ T cells (Fig. 6c) in patients were also observed. When we further analyzed double-positive populations, we found that patients had greater proportions of both Foxp3^+^CD25^high^ (Fig. 6d) and Foxp3^+^CTLA-4^+^ (Fig. 6e) Tregs than control subjects. We also assessed IL-10 expression in Foxp3^+^ CD4^+^ T cells. While some patients had increases of Foxp3^+^IL-10^+^ CD4^+^ T cells, there was no significant difference between patients and control subjects (Fig. 6f). Corticosteroid therapy did not significantly impact the percentage of Foxp3^+^IL-10^+^ CD4^+^ T cells (Fig. 6g). We also found that there was no significant difference in the amounts of IL-10 secreted by T cells from the different populations cultured for 36 h with αCD3/αCD28 beads (Fig. 6h). However, patient T cells secreted more Th2 cytokines, particularly IL-5 and IL-13, but not IFNγ. T cells from patients and control subjects secreted similar amounts of IL-21, IL-22, IL-17, and TNFα (data not shown). The percentage of Foxp3^+^IL-10^+^ cells in CD4^+^ T cells did not correlate with any of the clinical variables, including % predicted FEV1 and ACT scores (data not shown). However, the percentage of CTLA4^+^ cells in CD4^+^ T cells correlated with ACT scores (Fig. 6i), but not % predicted FEV1 (data not shown). This was not surprising because ACT scores do not always correlate with FEVs (Fig. 6j), as previously described [40].

We thus concluded that adult atopic asthma patients have an increase of circulating CD4^+^ T cells that are CD25^+^, FoxP3^+^, and CTLA-4^+^, FoxP3^+^CD25^high^, or FoxP3^+^CTLA-4^+^. In addition, the percentage of CD4^+^ T cells expressing surface CTLA-4 inversely correlates with asthma severity, as determined by the ACT scores. There was no significant correlation between the percentage of IL-10-expressing Tregs and clinical measurements.

### Altered expression of integrins on CD4^+^ and CD8^+^ T cells in atopic asthma patients

In addition to chemokine receptors, cell surface integrins expressed on T cells are also linked to T cell migration into lungs and could thus be involved in the pathophysiology of asthma [41–45]. We thus investigated whether T cells in atopic asthma patients have altered expressions of the integrins α4, β7, and CD11a that could be associated with asthma pathogenesis. The lack of α4 integrin not only impedes lymphocyte migration to lung and airways, but also prevents upregulation of vascular cell adhesion molecule-1 (VCAM-1) in inflamed lung vasculature [41, 46]. β7 along with α4 can also contribute to T cell and eosinophil accumulation in BAL and to airway inflammation in the absence of CCL19 and CCL21 [44, 45]. CD11a is an α chain of LFA-1 that is crucial for migration of leukocytes across the endothelial barrier into the surrounding tissues [47, 48].

**Fig. 7:**
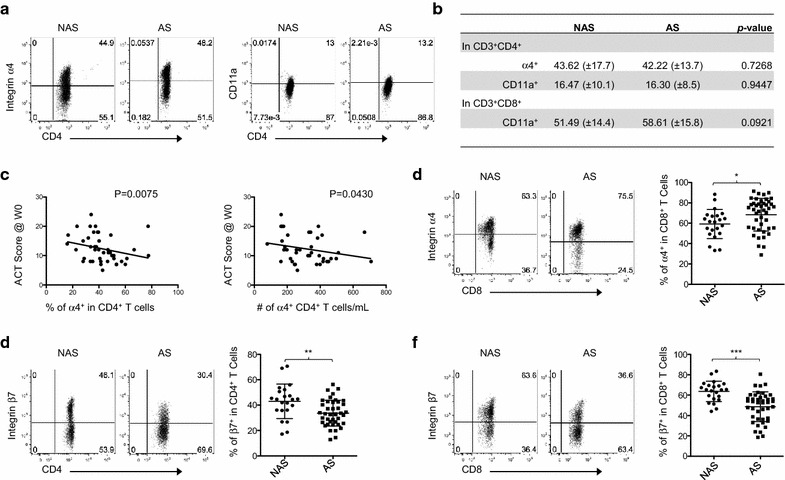
Frequency of α4^+^ CD4^+^ T cells in atopic asthma patients correlates with clinical severity. **a** Representative FACS analysis of whole blood staining for α4 and CD11a in CD4^+^ T cells in non-asthmatic subjects (NAS) and asthma patients (AS). Gates based on isotype antibody staining pattern. **b** Frequency analysis of integrin-expressing cells in CD4^+^ and CD8^+^ T cells in NAS and AS. **c** Correlation analysis of the frequency and number of α4-expressing CD4^+^ T cells in AS with ACT score. **d** Representative FACS data of whole blood staining (left) and compiled frequency of α4^+^ in CD8^+^ T cells (right) in NAS and AS. Representative FACS data of whole blood staining and compiled frequencies of β7-expressing CD4^+^ T cells (**e**) and CD8^+^ T cells (**f**) in NAS and AS. *P < 0.05, **P < 0.01, ***P < 0.001. Error bars indicate SD

Representative flow cytometry data show that patients and control subjects had similar percentages of α4^+^ (Fig. 7a, left) and CD11a^+^ CD4^+^ T cells (Fig. 7a, right). Summarized data of α4^+^ and CD11a^+^ CD4^+^ T cells are shown in Fig. 7b. Both patients and control subjects also had similar percentages of CD11a^+^ CD8^+^ T cells (Fig. 7b). Interestingly, however, the percentages and numbers of α4^+^ CD4^+^ T cells inversely correlated with the ACT scores (Fig. 7c). There was an increase of α4^+^ CD8^+^ T cells in patients (Fig. 7d), but the frequency of α4^+^ CD8^+^ T cells did not correlate with any of the clinical variables assessed, including ACT and % predicted FEV1 scores (data not shown). We also found decreases of β7^+^ CD4^+^ (Fig. 7e) and CD8^+^ T cells (Fig. 7f) in patients, compared to control subjects.

In conclusion, atopic asthma patients and non-asthmatic control subjects have a similar frequency of α4^+^ CD4^+^ T cells. However, their frequency of α4^+^ CD4^+^ T cells in patients inversely correlated with the ACT scores. Atopic asthma patients also have significantly lower percentages of CD4^+^ and CD8^+^ T cells that expressed β7 integrin, but not CD11a.

## Discussion

A persistent allergic inflammation in the lower airway may require an abundant presence of memory T cells [22, 23, 49] that can readily respond to allergens that are intermittently available throughout the year. In both murine models of allergic asthma and asthma patients, CD4^+^ memory T cells are involved in recurrent episodes of inflammation [23–25, 50]. Accordingly, we found a significant increase of circulating CD45RA^−^CD45RO^+^ memory CD4^+^ T cells in atopic asthma patients, compared to non-asthmatic control subjects. In this study, however, we further found that atopic asthma patients have a significant increase in memory CD4^+^ T cells that express CCR7, but not CCR7^−^ memory CD4^+^ T cells.

Both Tem and Tcm circulate in the blood. In contrast to Tem, CCR7^+^ Tcm cells can migrate to the lymph nodes and can quickly proliferate in response to infiltrating antigen-presenting cells (APCs). Thus, Tcm cells are also considered reactive memory cells [21, 51, 52]. They also can acquire an effector-like phenotype with the secretion of cytokines and chemokines [21, 52]. It is therefore possible that such long-lived CD4^+^ Tcm cells found in the blood of asthma patients could play an important role in the chronic inflammation in the lower airway in response to a variety of allergens that are intermittently available year-round. It was also important to note that the absolute numbers of CD4^+^ Tcm cells were also greater in atopic asthma patients than non-asthmatic control subjects. Therefore, the increase of CD4^+^ Tcm cells in atopic asthma patients was not due to a decrease of CD4^+^ Tem cells in their blood.

The roles of CCR4^+^ T cells in the pathogenesis of asthma are still controversial in both a murine model of asthma and asthma patients [29, 33, 43, 53–57]. The increase of CCR4^+^ CD4^+^ T cells in asthma patients has been previously reported [29, 33]. However, data from other studies indicate that the proportion of CCR4^+^ CD4^+^ T cells in peripheral blood or in the lungs does not always correlate with the severity of asthma [43, 57]. In our study, we found that atopic asthma patients have more circulating CCR4^+^ CD4^+^ T cells and this was mainly due to the increase of CD4^+^ Tcm cells. In line with this, the difference in the frequency of CCR4^+^ CD4^+^ T cells between atopic asthma patients and control subjects was even greater when we compared them in Tcm cells. The inverse correlation between the frequency of CCR4^+^ CD4^+^ Tcm cells and asthma severity further support that CCR4^+^ CD4^+^ Tcm cells could play an important role in the pathogenesis of atopic asthma. This increase of CCR4^+^ CD4^+^ Tcm cells can be seen across atopic asthma subtypes and severities. It was also important to note that current therapy (i.e. corticosteroids, β-agonists, leukotriene inhibitors, and combinations thereof) was not capable of reducing the frequency of either total CD4^+^ Tcm or CCR4^+^ CD4^+^ Tcm cells in atopic asthma patients. A previous study reported that corticosteroid treatment slightly decreased the percentage of CCR4^+^ total T cells, but it was performed with patients that had mild and stable asthma [58]. Our findings raise a fundamental question concerning the mechanisms responsible for the increased numbers of CCR4^+^ CD4^+^ Tem cells in atopic asthma patients. Nonetheless, our data might also be highlighting the possible reasons behind the ineffectiveness of current therapies (i.e., corticosteroids).

In line with the increase of CCR4^+^ T cells in patients, T cells from atopic asthma patients secreted more of IL-5 and IL-13 than T cells from non-asthmatic control subjects. Our data also show that there was no significant difference in the frequencies of CXCR3^+^ (for Th1), CXCR5^+^ (for Th21), or CCR6^+^ (for Th17) CD4^+^ T cells in the blood of patients and control subjects. Consistent with the similar frequencies of T cells expressing such chemokine receptors, T cells from patients and control subjects also secreted similar amounts of IFNγ, IL-21, IL-17, TNFα and IL-22.

Atopic asthma patients have a higher percentage of CRTH2^+^ cells, but this is only in the CD4^+^ Tcm cell compartment. Such increase in patients was not observed when we analyze the frequency of CRTH2^+^ cells in total CD4^+^ T cells or in total memory CD4^+^ T cells. This might explain inconsistent results from previous studies of the frequency of CRTH2^+^ cells in asthma patients [5, 35]. However, the increase of CRTH2^+^ CD4^+^ Tcm in patients was less significant than the increase of CCR4^+^ CD4^+^ Tcm cells. In addition, the frequency of CRTH2^+^ CD4^+^ T cells or CRTH2^+^ CD4^+^ Tcm cells did not show a significant correlation with any clinical variables, including ACT and % predicted FEV1 scores.

Consistent with the previously published data, we found that patients have an increased frequency of CD4^+^ Tregs as assessed by measuring the frequency of CD25^+/high^, Foxp3^+^, CTLA4^+^, Foxp3^+^CD25^high^, and Foxp3^+^CTLA-4^+^ CD4^+^ T cells [39]. Such increases in patients could be a natural process to counteract ongoing inflammatory responses, although corticosteroid treatment might also increase Treg frequency [59, 60]. However, the percentages of Foxp3^+^IL-10^+^ CD4^+^ T cells in the two groups of subjects were similar, and this is in line with the data from a previous study [61]. Only a few patients showed increased frequency of Foxp3^+^IL-10^+^ CD4^+^ T cells compared to other patients. The amounts of IL-10 secreted from T cells also showed a similar pattern to what was observed for the frequency of Foxp3^+^IL-10^+^ CD4^+^ T cells. This suggests that Tregs in asthma patients might not be fully functional, as previously reported [37–39]. We were not able to test the suppressive function of Tregs due to the limited amounts of blood samples collected from patients. The frequency of Foxp3^+^IL-10^+^ CD4^+^ T cells did not correlate with asthma severity (data not shown). Interestingly, we found that the frequency of CTLA4^+^ T cells correlated with the ACT scores. This suggested that fractions of Tregs in patients might still display certain levels of suppressive functions via the action of CTLA4, an inhibitory molecule, even though they may not be fully functional [39].

Integrins play key roles in adhesion of leukocytes to walls of blood vessels associated with inflammation and in migration of leukocytes to inflamed tissues [62, 63]. Integrins present on leukocyte surface belong to a large family of heterodimeric glycoproteins, which in the active conformation are composed of 2 noncovalently associated α and β subunits. Currently, 18 α and 8 β subunits are identified, which are associated in a restricted manner to create 24 heterodimers for specific ligand binding [64]. Among those, both α4 and CD11a, an α chain of LFA-1, are known to play important roles in leukocyte migration to lung [46–48, 65, 66]. A previous study also reported that IL-5 could increase VCAM-1 expression that can facilitate α4^+^ leukocyte migration to inflamed lung and airways [42]. One could thus expect an increase of α4^+^ CD4^+^ T cells in asthma patients. Interestingly, however, patients and control subjects have similar frequencies of circulating α4^+^ CD4^+^ T cells. However, we found that the frequency of α4^+^ CD4^+^ T cells significantly correlated with asthma severity, as assessed with the ACT scores. In contrast to α4^+^ and CD11a^+^ CD4^+^ T cells, patients have significant reductions in the percentages of β7^+^ CD4^+^ and CD8^+^ T cells. The clinical relevance of the decrease of β7^+^ T cells in asthma is not clear at this moment. β7 is generally known to play an important role in lymphocyte migration into guts [67, 68], and T cells in the lungs of asthmatic and non-asthmatic control subjects express only low level of α4β7 [43]. However, others have also reported that β7 along with α4 can contribute to T cell and eosinophil accumulation in BAL and to airway inflammation in the absence of CCL19 and CCL21 [44, 45].

## Conclusions

This study reports for the first time that an increase of long-lived CD4^+^ Tcm cells along with CCR4^+^ CD4^+^ Tcm cells is one of the major features of circulating blood T cells in adult atopic asthma patients. Our data also demonstrate that such T cell subpopulations seem to be resistant to current common therapeutics, including corticosteroids. Although atopic asthma patients have an increase of Tregs, these cells may not be fully functional, although CTLA-4 could contribute to their suppressive function. This study also provides evidence that the frequency of α4^+^ CD4^+^ T cells is clinically relevant in adult atopic asthma patients. Therefore, this study extends our knowledge on the pathogenesis of human atopic asthma and further guides us toward the rational design of therapeutics for atopic asthma in the future.

## Additional files

**Additional file 1: Table S1.** Antibodies used in this study.

**Additional file 2: Figure S1.** Correlation analysis of frequency of CD4+ and CD8+ memory T cells with age of recruited subjects.

## Abbreviations

ACT: asthma control test
AS: asthma patient
FEV1: forced expiratory volume in 1 s
NAS: non-asthmatic
Tcm: central memory T cells
Tem: effector memory T cells
