# Supplementary material for: Clinical implications of CD4+ T cell subsets in adult atopic asthma patients
Source: Allergy Asthma Clin Immunol. 2018 Mar 2;14:7. doi: 10.1186/s13223-018-0231-3 (PMC5833086; doi:10.1186/s13223-018-0231-3)
Supplement: Supplementary file 1 — Additional file 1: Table S1. Antibodies used in this study. [file 13223_2018_231_MOESM1_ESM.pdf]

Table S1. Antibodies used in this study

Marker	Clone	Company
CD3	UCHT1	BD
CD4	RPA-T4	Tonbo
CD8	RPA-T8	Tonbo/BD
CD45RA	HI100	BD
CD45RO	UCHL1	BioLegend
CXCR3	G025H7	BioLegend
CCR4	TG6/CCR4	BioLegend
CCR5	HEK/1/85a	BioLegend
CXCR5	51505	R&D Systems
CCR6	R6H1	eBioscience
CCR7	G043H7	BioLegend
CRTH2	BM16	BD
CD11a	MEM-25	ExBio
CD62L	DREG-56	BD
α4	9F10	BioLegend
β7	FIB504	BD
CD25	M-A251	BD
CTLA-4	14D3	eBioscience
IL-10	JES3-9D7	eBioscience
Foxp3	PCH101	eBioscience
Live/Dead Aqua		Invitrogen
mIgG2a	G155-178	BD
mIgG1	P3.6.2.8.1	eBioscience
mIgG2b	MPC-11	BIoLegend
mIgG1	MOPC-173	BD
mIgG2a	MOPC-173	BioLegend
rIgG2a	R35-95	BD
mIgG1	MOPC-173	BD
rIgG2a	R35-95	BD

m: mouse; r: rat
