# Supplementary material for: Clinical implications of CD4+ T cell subsets in adult atopic asthma patients
Source: Allergy Asthma Clin Immunol. 2018 Mar 2;14:7. doi: 10.1186/s13223-018-0231-3 (PMC5833086; doi:10.1186/s13223-018-0231-3)
Supplement: Supplementary file 2 — Additional file 2: Figure S1. Correlation analysis of frequency of CD4+ and CD8+ memory T cells with age of recruited subjects. [file 13223_2018_231_MOESM2_ESM.pdf]

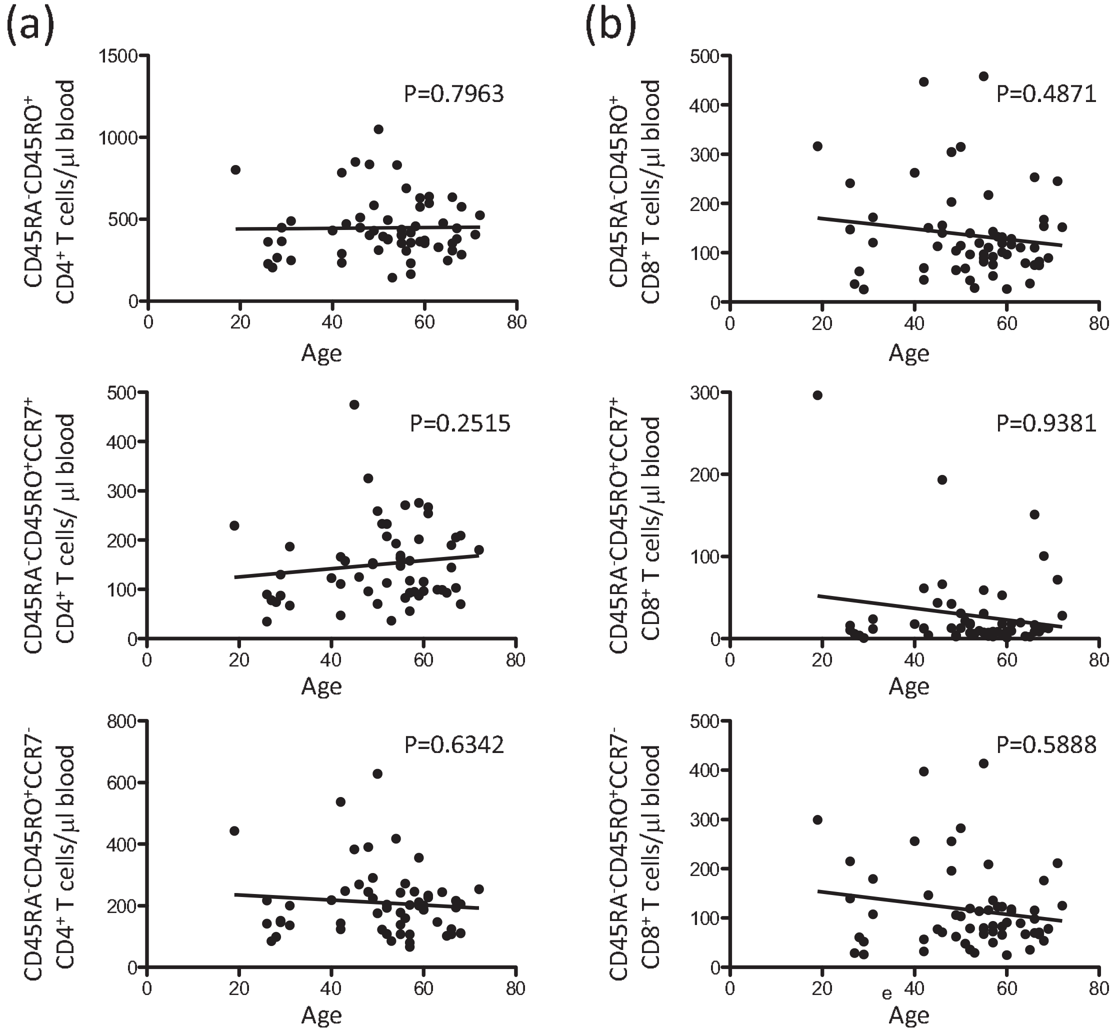

Fig. S1 Correlation analysis of frequency of $CD4^+$ and $CD8^+$ memory T cells with age of recruited subjects. The numbers of $CD45RA^-CD45RO^+$, $CD45RA^-CD45RO^+CCR7^+$, and $CD45RA^-CD45RO^+CCR7^-$ $CD4^+$ (a) and $CD8^+$ T cells (b) were enumerated per mL whole blood utilizing counting beads. Cell numbers were assessed for correlation with recruited subjects' age utilizing non-parametric Spearman correlation.
